# Biosensors for the Rapid Detection of Cardiovascular Biomarkers of Vital Interest: Needs, Analysis and Perspectives

**DOI:** 10.3390/jpm12121942

**Published:** 2022-11-22

**Authors:** Laure Abensur Vuillaume, Justine Frija-Masson, Meriem Hadjiat, Thomas Riquier, Marie-Pia d’Ortho, Pierrick Le Borgne, Christophe Goetz, Paul L. Voss, Abdallah Ougazzaden, Jean-Paul Salvestrini, Thierry Leïchlé

**Affiliations:** 1 Emergency Department CHR Metz-Thionville, 57000 Metz, France; 2 IRL 2958 Georgia Tech CNRS, 57000 Metz, France; 3 Digital Medical Hub, AP-HP, 75000 Paris, France; 4 Service de Physiologie Clinique-Explorations Fonctionnelles, Hôpital Bichat, AP-HP, F-75018 Paris, France; 5 UFR de Médecine, Université Paris Cité, NeuroDiderot, INSERM, F-75019 Paris, France; 6 Biology Department, CHR Metz-Thionville, 57000 Metz, France; 7 Emergency Department, Hôpitaux Universitaires de Strasbourg, 67000 Strasbourg, France; 8 INSERM (French National Institute of Health and Medical Research), UMR 1260, Regenerative NanoMedicine (RNM), Fédération de Médecine Translationnelle (FMTS), University of Strasbourg, 67000 Strasbourg, France; 9 Clinical Research Support, CHR Metz-Thionville, 57000 Metz, France; 10 Georgia Institute of Technology, School of Electrical and Computer Engineering, Atlanta, GA 30332-0250, USA; 11 LAAS-CNRS, 31400 Toulouse, France

**Keywords:** cardiac biomarkers, biosensors, troponin, BNP, D-dimers

## Abstract

We have previously surveyed a panel of 508 physicians from around the world about which biomarkers would be relevant if obtained in a very short time frame, corresponding to emergency situations (life-threatening or not). The biomarkers that emerged from this study were markers of cardiovascular disease: troponin, D-dimers, and brain natriuretic peptide (BNP). Cardiovascular disease is a group of disorders affecting the heart and blood vessels. At the intersection of medicine, basic research and engineering, biosensors that address the need for rapid biological analysis could find a place of choice in the hospital or primary care ecosystem. Rapid, reliable, and inexpensive analysis with a multi-marker approach, including machine learning analysis for patient risk analysis, could meet the demand of medical teams. The objective of this opinion review, proposed by a multidisciplinary team of experts (physicians, biologists, market access experts, and engineers), is to present cases where a rapid biological response is indeed valuable, to provide a short overview of current biosensor technologies for cardiac biomarkers designed for a short result time, and to discuss existing market access issues.

## 1. Introduction

Cardiovascular diseases are a group of disorders affecting the heart and the blood vessels. These disorders mainly include heart diseases that affect the vessels or the structure of the heart (coronary, congenital, hypertensive) and thromboembolic diseases (deep vein thrombosis and pulmonary embolisms). These pathologies expose patients to risks of acute or chronic complications such as acute coronary syndrome (ACS), chronic or acute heart failure (HF), chronic kidney disease (CKD) and ultimately, death. These disorders are the leading cause of death in the world, and as a result, many clinical researchers are interested in improving the overall management and treatment of these conditions. One of the means to achieve this goal is to provide accurate and rapid diagnosis through the support of biological analysis. 

However, it seems that the need for medical diagnostic tools is not always satisfied by the current technologies available on the market [1]. To better understand this situation, we wanted to take stock of the priority needs in medical diagnosis using biomarkers. Our team recently surveyed a panel of 508 physicians around the world regarding biological markers that would be relevant in medical practice if obtained in a very short time frame. This study, which was recently published, showed the interest of physicians in speeding up the process of obtaining biological results, especially in the case of cardiovascular biomarkers [1]. Indeed, the biomarkers that stood out from this study, i.e., the most cited ones, were Troponin (a 95% CI of 51% (46.24, 54.94)), D-dimers (a 95% CI of 29% (24.80, 32.68)) and Brain Natriuretic Peptide (BNP; a 95% CI of 13% (10.25, 16.13)), all markers of cardiovascular diseases. In this study, physicians reported that a faster biomarker result would lead to “time savings, fluidity for hospital or a gain in private practice,” improvement such as “guiding a course of action, helping to make a therapeutic decision, avoiding unnecessary treatment while waiting for a result, and avoiding diagnostic delay,” and faster referral of patients to an appropriate facility. 

Here, we review these markers, and we discuss the clinical limits of their use in light of our multidisciplinary experience. We also briefly present the few recent technological developments in biosensors that indeed achieve a short analysis time for these specific biomarkers and discuss the main obstacles to the commercial success of such biosensors.

## 2. Cardiovascular Biomarkers of Interest Identified by Physicians 

### 2.1. Troponin

#### 2.1.1. Structure

Troponins are structural proteins of the myocardial contractile system. The troponin complex consists of three subunits: (1) Troponin T (TnT) is a subunit binding to tropomyosin and, therefore, responsible for muscle contraction; it is present in cardiac muscle and striated muscle. TnT has three isoforms coded by different genes. (2) Troponin I (TnI) is also involved in the control of myocardial contractility by allowing muscle relaxation. It binds to tropomyosin, actin, TnT, and Troponin C. TnI also has three isoforms with specific peptide sequences. (3) Troponin C (TnC) enables the binding of calcium and magnesium, both necessary for muscle contraction. It has only one isoform common to all striated muscles and is, therefore, of no interest in cardiology because it is not specific to the myocardium.

#### 2.1.2. Clinical Relevance

Only the cardiac isoforms of troponins I and T are of interest in cardiology. During myocardial necrosis (infarction), these troponins are released into the bloodstream during cell lysis. They are, therefore, an indirect reflection of myocardial suffering during an infarction. They are currently the most sensitive and specific markers of myocardial damage and represent the “gold standard” for the diagnosis and prognosis of ACS. In the clinical setting, Troponin T has higher sensitivity values and is the one that is most commonly measured. The universal definition of myocardial infarction includes troponin elevation with at least one value above the 99th percentile, associated with evidence of cardiac ischemia (symptoms, electrocardiogram (ECG) changes or imaging) [2]. When there is an obvious ECG change associated with typical pain for more than 20 min (ACS with ST-segment elevation on ECG: ACS ST+ or STEMI), the main element of management is revascularization without the need to wait for a biological result. Troponin is of no interest in clinically evident infarctions or in symptomatic patients without obvious electrical changes but with a very high vascular risk. It is relevant in cases where pain is not associated with an ECG change in patients at medium or low risk, who nevertheless have a true subclinical infarction (ACS without ST-segment elevation on the ECG: ACS ST- or Non-STEMI) [3]. Note that patient risk stratification is performed using the GRACE score (calculated from Killip class, systolic blood pressure, heart rate, age, serum creatinine, history of cardiac arrest on admission, ST-segment change or not, and cardiac enzyme increase) [4]. Nevertheless, troponin elevation is not specific to ACS and can be observed in other cardiac or pulmonary diseases [5,6]: severe arrhythmia, acute pulmonary edema, pulmonary embolism, severe decompensation of chronic obstructive pulmonary disease, severe anemia, severe arterial hypertension, non-ischemic myocardial damage (myocarditis, cardiac contusion), stroke, or sepsis. Thus, in the case of ST- or non-STEMI ACS, it is recommended to obtain two high-sensitive Troponin assays at 3-h intervals in order to confirm or deny a 30% increase in values between the two assays [7]. However, an immediate elevation of troponin to values >99th percentile compared with a healthy population may suggest an infarction and should require immediate investigation.

#### 2.1.3. Biological Assay

A patient with a healthy heart usually has a troponin level of zero. Recent ultrasensitive assays detect troponin levels of 2 pg/mL. The ultra-sensitive assays for the two cardiac isotopes I and T are based on the same type of analysis: the ELISA immunoassay. The choice of I or T assay is organizational; each standard is type-specific, and there is no equivalence between the two assays. These tests are performed by a medical laboratory within a median time of one hour (recommended time), including an incompressible 18-min on-site analytical cycle (which may vary depending on the automated system) [8]. The sample is analyzed from a dry tube, EDTA or lithium heparin, preferably taken without a tourniquet and after purging the sampling tube, thus limiting hemolysis due to the addition of air, which can falsely increase the result; the tube, with a volume of 4 mL to 7 mL, must be filled to 90% to avoid the formation of fibrin [9]. The anticoagulant potentially used in the tube prevents the coagulation cascade inside the tube. It allows the biologist to study the plasma after immediate centrifugation of the sample and thus reduce the analysis time (since it would otherwise be necessary to wait for the blood to coagulate before centrifuging, and this “natural” coagulation could possibly create interferences via the fibrin). The production cost of analysis in a large laboratory is approximately 2 euros, excluding the cost of personnel and premises. 

The characteristics of the Troponin biomarkers are summarized in Table 1.

### 2.2. D-Dimers

#### 2.2.1. Structure

D-dimers are breakdown products of fibrin, which is the main component of a blood clot. Thus, the existence of D-dimers in the blood is an indicator of the existence of a blood clot. When a clot forms in the blood, thrombin is formed. Thrombin releases 4 fragments from the fibrinogen molecule: two fibrinopeptides A and two B. The fibrinogen molecule thus becomes a fibrin monomer. This fibrin monomer polymerizes to become a soluble fibrin polymer with a fragile state. This polymer is stabilized and becomes insoluble thanks to the coagulation factor XIIIa, activated by thrombin. The presence of this stabilized polymer will trigger a fibrinolysis cascade involving plasmin, a proteolytic enzyme. This enzyme cuts the insoluble fibrin, which results in the appearance of D-dimers as degradation products.

#### 2.2.2. Clinical Relevance

D-dimer levels are elevated in plasma in the presence of acute thrombosis due to the simultaneous activation of coagulation and fibrinolysis. The sensitivity of D-dimers in clinical practice is in the range of 90 to 100%, with an excellent negative predictive value. Hence, the diagnosis of thrombosis can be excluded in the case of a normal value, making the diagnosis of deep vein thrombosis or pulmonary embolism unlikely [10]. Nevertheless, the positive predictive value of an elevated D-dimer level is relatively low, mainly because the D-dimer level is increased in many clinical situations (cancer, pregnancy, chronic inflammatory disease, sepsis, and COVID-19). Thus, the clinical practice, codified by international recommendations in 2019 [10], is first guided by a pre-test probability level (Wells score [11,12]), associated with a biological test validated in terms of diagnostic performance (tests with diagnostic sensitivity ≥95% (strong agreement, recommendation grade A). Thus, a normal test and a low clinical probability enable to stop further paraclinical investigations. Conversely, in patients with an abnormal result, imaging of the lungs or deep veins should be conducted, and treatment should be started if the delay before imaging is significant. 

#### 2.2.3. Biological Assay

D-dimer is one of the most interesting and studied biomarkers for thrombosis. The analysis is performed on whole blood or plasma. In whole blood, the D-dimer stability is 24 h at room temperature or refrigerated, while in plasma, samples can be stored for 2 years in a freezer at −24 °C or −74 °C. Routine analyses are performed on plasma after centrifugation. Routine analyses are performed by ELISA methods or so-called “quantitative latex” techniques (of sensitivity comparable to that of ELISA). Quantitative latex is an immunoassay using latex particles coupled to an anti-D-dimer monoclonal antibody allowing a precise quantitative assay: the antigen-antibody reaction between the D-dimers and the monoclonal antibodies covalently attached to the latex microspheres leads to agglutination of the microspheres, which induces an increase of the turbidity of the reaction mixture, thus an increase of the absorbance of the medium that depends on the D-dimer concentration. However, there is currently no standardization of D-dimer assay techniques. Biological standards, therefore, differ depending on the technique used and each test manufacturer must provide its own standard. In current practice, most of the tests, especially those used in the new generation of ELISA tests, adopt standards of test negativity for values < 500 ng/mL and even clinical thresholds below 1000 ng/mL with the new PERC/YEARS criteria with age adjustment [13]. 

The characteristics of the D-dimer biomarkers are summarized in Table 1.

### 2.3. BNP/NT-ProBNP

#### 2.3.1. Structure

Brain natriuretic peptide (BNP or B-type natriuretic peptide) is a molecule belonging to the family of natriuretic peptides. This family also includes ANP, CNP, DNP and urodilatin, which currently have no clinical implications [14]. BNP in humans is predominantly synthesized by cardiomyocytes [15]. BNP is a peptide comprising 32 amino acids, 17 of which form a ring closed by a disulfide bridge [15]. It is synthesized as a pre-proBNP protein comprising 134 amino acids and secondarily cleaved into proBNP (108 amino acids). The latter is equimolarly cleaved into BNP and N-part (NT-pro-BNP, 76 amino acids) after a maturation phase. 

#### 2.3.2. Clinical Relevance

BNP is synthesized in response to increased ventricular pressure or a volume overload. It counteracts the increase in ventricular pressure and volume overload by inducing peripheral vasodilatation, increasing glomerular filtration, decreasing renal sodium reabsorption, and inhibiting the Renin-Angiotensin-Aldosterone system. NT-proBNP alone has no recognized clinical action to date, but its measurement is used as a reflection of BNP synthesis. This characteristic makes it a sensitive marker in clinical practice in chronic and acute heart failure. Nevertheless, this marker is not specific and is also increased in many acute and chronic diseases: acute and chronic pulmonary pathology with right ventricular repercussions, valvular heart diseases, primary and secondary left ventricular hypertrophy, renal failure, atrial arrhythmia, sepsis, acute myocardial ischemia, chronic systolic dysfunction, hyperthyroidism, Cushing’s disease or glucocorticoid use, primary hyperaldosteronism, Addison’s disease, diabetes mellitus, cirrhosis with ascites, paraneoplastic syndrome, and subarachnoid hemorrhage [16]. BNP and NT-proBNP are deemed useful to diagnose chronic cardiac failure [17] but should be included in a global algorithm comprising clinical evaluation and other paraclinical exams. A normal dosage has a high negative predictive value, thus rendering chronic cardiac failure improbable [18,19]. An elevated dosage requires further cardiac investigations [18]. It should be noted that these two markers are not, to date, recommended for screening in a healthy population [17]. Neither is it recommended for therapeutic adjustment in diagnosed patients [17]. BNP and NT-proBNP are, however, very useful in patients with dyspnea to differentiate cardiac failure from respiratory diseases [20]. Their values increase with the severity of cardiac failure. National and international recommendations include BNP or NT-proBNP dosage when assessing a patient with acute dyspnea [19,21]. 

#### 2.3.3. Biological Assay

Brain natriuretic peptide (BNP) and the N-terminal fragment of proBNP (NT-proBNP) are cleavage products of pro-BNP. The half-life of NT-proBNP in the blood is longer: 60 to 120 min versus 20 min for BNP. There is no international standard of measurement for these biomarkers [16]. Historically, natriuretic peptides have been determined by methods using iodine 125. Nowadays, the so-called “cold” ELISA methods based on non-radioactive tracers are routinely used [22,23]. The NT-proBNP assay uses the same capture antibodies (polyclonal or monoclonal antibodies) and the same calibrator (Roche license). The BNP assay, on the other hand, is less standardized and uses several pairs of antibodies, which can give variable results for the same sample and must therefore take into account the measurement method. The coagulation cascade does not degrade NT-proBNP but may influence BNP. The NT-proBNP assay can therefore be performed on serum in a dry tube or on dry plasma in lithium heparin, di- or tripotassium EDTA tubes. The NT-proBNP sample has good stability at room temperature for 7 days and 10 days at 4 degrees. Conversely, the BNP sample degrades under the action of blood proteases with a decrease of rate at 4 h [24]. The sample must be frozen quickly to ensure the stability of the assay and the collection in EDTA tubes. Thus, the NT-proBNP assay is comparable from one laboratory to another. Its determination is stable over time in the sample. In addition, NT-proBNP has less intra-individual variation. As explained above, NT-Pro-BNP reflects BNP concentration, which implies that these two biomarkers have the same clinical significance and that neither is superior to the other [17]. However, they are not comparable with each other for the reasons mentioned above (different assay methods). The standards depend on the technique and increase with age. They depend on the sex (female > male), the body mass index (lower plasma BNP concentration in obese patients) and the glomerular filtration rate [25]. In acute heart failure, the thresholds to be considered negative (high probability 95% CI) are <100 pg/mL for BNP and <300 pg/mL for NT-proBNP. The thresholds to be considered positive (high probability) are >400 pg/mL for BNP and, depending on the age, >450 pg/mL, >900 (50–75 years), and >1800 (>75 years) for NT-proBNP. Between these two types of threshold, there is a gray area where it is not possible to have a clear answer, i.e., rule out or diagnose heart failure.

The characteristics of NT-ProBNP biomarkers are summarized in Table 1.

## 3. Should Biological Results for These Markers Be Accelerated?

Despite a need expressed by the panel of physicians, rapid access to a troponin result is questionable, at least in the emergency field. Indeed, in typical cases (pain and ECG), troponin has no interest, and in less typical cases, it is necessary to evaluate the kinetics between two samples [1]. If the kinetics are positive, revascularization must take place within 24 h; the patient is then either hospitalized or discharged home after a minimum stay of about 4 h in the emergency room. Thus, a rapid result is of little benefit to the patient if he or she is in the emergency department since the patient is being monitored. The waiting time for the second test result could be shortened, but the development cost/gain balance of less than one hour is not positive from a medico-economic point of view. The question of a rapid test can be raised in a general practitioner’s or cardiologist’s office who would like to have a diagnostic orientation for a patient. However, the turnaround time for a troponin test in a hospital laboratory is, at best, 4 h between the sampling and the result. In addition, in this context, the performance of a biological test, if positive, outside of the hospital may represent a real loss of opportunity for the patient. If it is negative, it does not formally permit the elimination of an ischemic phenomenon since only the kinetics provide an answer.

When the test is requested appropriately, obtaining a rapid result for D-dimer is relevant in the office or the hospital setting. If the probability of thrombosis is low or moderate, a negative result allows the diagnosis of thrombosis or pulmonary embolism to be ruled out and to focus on other etiologies. In general practice, in the absence of an immediate response to the suspicion of deep vein thrombosis, this suspected diagnosis of severity leads to the initiation of presumptive treatment with anticoagulants while awaiting the biological and/or ultrasound results. In the case of a suspected pulmonary embolism, the general practitioner refers the patient to the emergency room to confirm or rule out the diagnosis. Also, in the hospital setting, performing this test quickly would allow the patient to be referred more quickly. Nevertheless, the limit of a rapid test lies in its sensitivity, which must be at least ≥95% to allow perfect integration into the current and future recommendations. 

An erroneous diagnosis is made in approximately 20% of dyspneic patients at the time of emergency department discharge [26], inducing an excess in mortality related to the non-recognition of acute heart failure, which is probably avoidable. Indeed, in acute dyspnea, the capacity to clinically differentiate acute heart failure from another etiology is limited [20]. The existence of associated pathologies resulting in dyspnea is found in 70% of cases in the literature [27,28], which most often results in an erroneous or underestimated diagnosis and delayed therapy. Pre-hospital management of acute heart failure with early drug treatment, especially in critical patients, is associated with better patient survival [29]. However, the use of these therapies seems to increase the mortality of patients misdiagnosed at this initial phase [29], even though the literature is still divergent. A rapid and reliable determination of BNP or NT-proBNP in a few minutes would be relevant in clinical practice for acute heart failure situations, starting in the pre-hospital setting [30]. A specific treatment administered early on would bring a benefit in terms of patient morbi-mortality. The dosage could be coupled with the use of ultrasound, which is very relevant in the recognition of cardiac congestion and could be integrated into a machine learning tool to increase diagnostic accuracy [31].

## 4. Development of Diagnostic Tools with a Shorter Time to Result

### 4.1. Biosensors for Cardiac Biomarkers

A biosensor is defined by the International Union of Pure and Applied Chemistry (IUPAC) as a self-contained integrated device capable of providing analytical information by using a biological recognition element (biochemical receptor) in direct spatial contact with a transduction element [32]. In addition, the IUPAC recommends distinguishing a biosensor from a bioanalytical system that requires additional processing steps to obtain the analytical information. Biosensors have been the subject of intense research efforts in recent years for environmental and health applications, where biomarkers are used for diagnostic or prognostic purposes. Thus, many biosensors have been proposed for the determination of cardiac biomarkers and have been the subject of recent reviews [33,34]. Enzymes, antibodies, oligonucleotides, aptamers and even membrane receptors and whole cells can be used as biochemical receptors, but in the case of cardiac biomarker detection, troponin, d-dimers and BNP are usually recognized using specific antibodies (as in the ELISA assay). Various transduction methods are used to turn the biorecognition event into a measurable physical signal [35], most commonly electrical (e.g., where transistors or nanowires are used to measure the electrical charges of the captured biomarker), mechanical (e.g., relying on a mechanical resonator which resonant frequency depends on the mass added onto the sensor, as in the quartz microbalance system), or optical (e.g., where a change in the index of refraction of the bioactive layer induced by the presence of the biomarker is measured, as in surface plasmon resonance setup). All transduction mechanisms have been explored in the design of biosensors for cardiac biomarkers, but most of them rely on electrochemical and optical transduction methods, historically the most used and studied transduction mechanisms. Because biosensors are based on micro- and nanotechnologies, they are easily miniaturized and could be used for point-of-care analysis, where a portable instrument performs the analysis at the patient’s bedside. However, a biosensor is, by definition, an analytical method that can result in instruments of different formats: many biosensors, delivered as benchtop equipment, are not necessarily portable. Biosensors achieve lower detection thresholds, thus offering promising alternatives to currently used bioanalytical gold standards techniques, such as ELISA methods conducted with automated central laboratory platforms [36]. However, when we look at the literature of the last 5 years, almost 200 studies dealing with biosensors for troponin have been published (194), with about 50 concerning BNP/NT-proBNP and only eight for D-dimer (source: Web of Science). It is important to mention that the portable sensors on the market do not currently offer D-dimer measurements. These elements suggest that the community of engineers, chemists, and physicists who develop these sensors are also convinced of the usefulness of quickly measuring troponin, which is debatable in view of the elements presented above. Besides, a review of biosensors developed recently shows that other biomarkers that were not listed by the panel of physicians, e.g., C-reactive protein, which is an established cardiac biomarker, are targeted by the sensor community [33].

BNP/NT-proBNP and D-dimer are relevant markers that could have a real impact if quickly measured. However, are the sensors presented so far in the literature able to provide a rapid response? If so, could they provide a viable technological answer for the democratization of point-of-care, off-site medicine? To answer this question, it is important to define the specifications required for this application. It is now generally accepted that an ideal test for bedside use should meet the criteria known as ASSURED—recently revised to REASSURED [37]. These are R for Real-time connectivity, E for Ease of specimen collection, A for Affordable, S for Sensitive (avoid false negatives), S for Specific (avoid false positives), U for User-friendly, R for Rapid and robust, E for Equipment free and D for Deliverable to end-users. In the specific case of sensors dedicated to the diagnosis of cardiovascular diseases, some of these criteria (which have been established for sensors dedicated to guiding treatment decisions and clinical management of infectious tropical diseases and sexually transmitted infections in developing countries) are more or less valid and important. In addition, we can further specify some of these criteria in light of the current recommendations and the constraints imposed by off-site use, such as community settings.

Thus, it is, of course, necessary that the proposed sensor offers at least a limit of detection (LOD) below the thresholds of negativity which are at the level of 0.1 ng/mL and 500 ng/mL for BNP and D-dimer, respectively: these limits of detection are largely attainable for sensors based on new technologies [38], using electrical [39], mechanical [40], or optical [41] transduction methods. It is interesting to note that Troponin, which appears to be the least relevant of the markers, is the one that requires the highest LOD. There are currently many means of transduction used for the determination of BNP [42]; however, few solutions are proposed for the determination of D-dimers [43]. The very nature of D-dimers, which are heterogeneously made up of fragments produced throughout the fibrinogen degradation process and which therefore do not have a precise molecular weight, makes their determination more complicated to implement [43]. This probably explains the lack of papers found on D-dimer biosensors in the literature. Among the plethora of biosensors that have been developed for cardiac biomarkers, only a few examples can be found for the rapid detection of BNP/NT-ProBNP and D-dimer that could answer the need expressed by the panel of physicians, which indicates that there is room for improvement and a lot of work to be done in the sensor community. A summary of the characteristics and performances of these biosensors is provided in Table 2.

### 4.2. Marketed Biosensors

There are commercial portable or benchtop devices based on biosensors that allow automated measurement of troponin and BNP/NT-proBNP from a few drops of the sample (between 10 µL and 200 µL of whole blood or plasma) typically in less than 10 min, hence satisfying the criteria of portability and fast response [34]. Portable devices such as the iSTAT or the Minicare I-20 are based on different detection methods (two-site ELISA and amperometric reading in the first case, immunological reaction on magnetic beads coupled with optical detection in the second case) using advanced technologies such as microfluidics and silicon electrochemical micro-sensors [48,49]. These devices are based on the use of a disposable cartridge coupled with a connected reader. Although these devices are much less expensive than the analytical systems used in centralized laboratories (which nevertheless offer the possibility of processing a large number of samples), they still require significant investment: indeed, although the cost of a cartridge for a single test is in the region of 10 euros, the cost of the reading device is rather in the range of 10–20 k€. These commercial devices could, therefore, theoretically meet the need raised by our study to obtain these cardiac biomarkers quickly. However, in view of the answers provided in our survey, it seems that these devices are not used routinely and that there are, therefore, obstacles to their use [1]. Besides, these devices are not currently included in the guidelines for troponin determination in acute pathology [50].

### 4.3. Obstacles to the Use and the Commercialization of Biosensors in Medical Setting for Cardiac Markers

The obstacles to the use and implementation of biosensors in routine medical practice are the following: (1) high cost and financial pressure, (2) habits and recommendations of good practice, (3) expected performance, (4) obstacles to innovation, and (5) constraints related to the sample—the high cost of implementation and financial pressure being the most important obstacle.

#### 4.3.1. High Cost and Financial Pressure

As mentioned above, the determining factor that has not allowed portable commercial technologies (which meet all the other criteria) to become established in medical practices is the overall cost of the technique (cost of the device, consumables, maintenance), which must be set compared to the cost of the procedure itself. Analytical devices based on very low-cost technologies, such as paper-based fluidics inspired by lateral flow assays, are very promising in this sense, even if they still lack sensitivity and the colorimetric reading remains semi-qualitative [51]. Microelectronic sensors promise to be inexpensive. However, this is true if the chips are produced in large volumes, as for consumer electronics. It is moreover obvious that it is necessary to ensure the industrialization of the sensor: from this perspective, the promising biosensing technology based on surface functionalization and electronic detection using silicon has a head start on the emerging approaches based on high-performance two-dimensional carbon material (graphene). Moreover, and this remark is valid whatever the transduction method, the type of bioreceptor and its immobilization method on the sensor surface can greatly influence the final cost of the consumable: for example, the use of aptamers or molecularly imprinted polymers would reduce the cost of immunological sensors that classically rely on the use of antibodies [34]. The cost related to the implementation and the use of biosensors means that small hospitals are not likely to make the integration of new tools and technologies profitable. In the community, this cost might represent too high of an investment for a single doctor, especially since the procedure is not currently valued. At the same time, financial pressure on health systems has also affected medical laboratories, which have been forced to group together in order to reduce costs and improve efficiency. More stringent quality and production standards have been added, which puts pressure on the available capital of companies operating medical laboratories, leading to a reduction in territorial coverage and specialization of structures, which has resulted in longer delays in the delivery of results. Other more complex and contextual elements must also be taken into account; cost is certainly a major obstacle initially, but also later on—the cost of reagents and the cost of maintenance without possible invoicing are to be taken into account. Other elements to take into account include the time it takes to carry out the analysis, taken during a consultation, and to maintain the equipment (the maintenance of the equipment intended for the measurement of biomarkers is defined by regulation and is enforceable against the health professional who uses it) and the problem of the reliability of the results if the equipment is poorly managed or poorly maintained, as well as the availability of human resources to conduct these tasks.

#### 4.3.2. Habits and Integration into Good Practices

The second obstacle is related to the habits of physicians. Community physicians do not usually perform analyses on their patients themselves. In France, in the city as in the hospital, point-of-care biology is the responsibility of a biologist, where the role of the biologist is one of responsibility, monitoring, control and validation. Hence under the current regulations, a physician would require the help of a biologist in their office, which is virtually impossible [52]. 

In addition, in order to be followed by the medical profession, these new technologies must be integrated into the recommendations of good practice.

#### 4.3.3. Performances

The technical performances of the biosensors, e.g., LOD, have been discussed, but ultimately, reliability in terms of sensitivity/specificity is what matters, as it may result in a loss of chance of survival for patients in certain situations. It is thus essential that these innovations be efficient in order to replace laboratory analyses.

#### 4.3.4. Barriers to Innovation

There are also obstacles to innovation due to a lack of scientific proof and a lack of knowledge of the processes. Indeed, the process of technology transfer, then validation and marking by the European Community or FDA, can be a major obstacle due to a lack of knowledge of the system. It is important to be accompanied and guided throughout these steps.

#### 4.3.5. Constraints Related to the Sample

The type of sample and its processing is a central element to be taken into account, which explains in large part why current techniques are under the responsibility of a biologist and cannot, therefore, allow for delocalized medicine. Ideally, the same model should be followed as for blood glucose testing with finger prick sampling, which allows for the simple collection of the necessary volume for analysis by miniaturized sensors. However, threshold analyses for finger prick testing, which are not the same as for venous testing, must be established.

### 4.4. Perspectives

#### 4.4.1. Tools for Faster Analysis

A fundamental criterion at the center of our study [1] is the response time of the analytical method: it corresponds to the time between the sample being taken and the moment when the result is published. For this response to be fast (<5 min), it is obvious that the measurement must be performed on-site, so the technique must be at least integrated, if not portable. Also, the response time includes time dedicated to sample preparation; hence, given the response kinetics of biosensors that are capable of detecting ng/mL biomarkers in a few minutes for a sample volume of a few µL [38], it is highly desirable not to have to perform sample preparation before the analysis so techniques that require incubation, centrifugation, or the addition of additives should be avoided. In this sense, detection methods without labeling or amplification that analyze crude samples are the most promising [53].

#### 4.4.2. Tools Centered on Biology

At a time when biology labs have been grouped together and doctors need increasingly rapid turnaround times to make their decisions, it is essential to reflect on how physicians and biologists can work together to deliver faster analysis at the patient’s bedside. To this aim, tele-expertise is a relevant approach to consider. It would be “easy” for clinicians to take a shortcut and ignore biologists, but it makes much more sense, in order to respect the healthcare ecosystem, including the economy, to fully integrate them into the process. Some technologies, for example, in France, are trying to distinguish themselves, such as the Diapason device, which performs INR (coagulation biomarker for measuring the effectiveness of anti-vitamin K drugs) at the bedside of patients, with results delivered by remote expertise from local biologists [54]. 

#### 4.4.3. Towards Multi-Marker Analysis

In a purely exploratory but technologically feasible way, which would probably allow the new technologies to be useful to physicians and to justify an additional cost, is the multi-marker approach, i.e., the possibility of measuring numerous markers in a single sampling and a single measurement procedure (contrary to the portable commercial systems which propose a cartridge per marker). This would make it possible to respond to the market for troponin and to meet the reality of the recommendations for other markers (here, D-dimers and BNP). There are now multiple examples of research works following the promising path of multi-markers analysis, even if D-dimers are still not targeted as a biomarker, as very recently reviewed [55].

## 5. Conclusions

At the intersection of medicine, basic research and engineering, biosensors could have a prominent place in the hospital or in the primary care ecosystem today. Cardiovascular disease is a public health priority and is widely recognized by the international panel of physicians we interviewed about the importance of obtaining bioanalytical results faster than with standard centralized laboratory techniques. This panel highlighted cardiovascular markers as a priority. Rapid, reliable, and inexpensive bioanalysis with a multi-marker approach, including machine learning analysis, could meet the demand of medical teams. However, it is important to overcome existing technological barriers by implementing a multidisciplinary approach from the outset and considering specific market access issues.

## Figures and Tables

**Table 1:** Characteristics of the cardiac biomarkers cited by an international panel of physicians, as obtained from central laboratories.

Biomarker	Sample	Tube	Sample Treatment	Reference Analytical Method	Cut-Off Value	Detection Limit	Total Time of Analysis (Excluding Routing)
Troponin hs	Peripheral venous blood Serum Plasma	Dry tube Dipotassium EDTA Tripotassium EDTA Lithium sodium heparin	No	Elisa (method related to an international reference standard) troponin T or I (non-standardized methods) Example: ECLIA determination on Cobas 6000	Baseline < 14 ng/L	5 ng/L (limit of quantification 13 ng/L)	Analytical cycle (18 min) without pre-analysis
D-dimer	Peripheral venous blood (total or serum)	Citrate tube	The sample must be analyzed within 8 h	2nd generation agglutination technique using a suspension of latex microparticles on which are fixed monoclonal antibodies specific to D-dimers	Baseline < 500 ng/mL	270 ng/mL (linearity zone: 270–20,000 ng/mL)	Analytical cycle (18 min) without pre-analysis
NT-Probnp	Peripheral venous blood Serum Plasma	Dry tube with or without separating gel EDTA dipotassic EDTA tripotassic with or without separating gel	Ambient temperature	ECLIA	Baseline >350 ng/L if under 50 years old >450 ng/L if 50–75 years old >950 ng/L if >75 years old	10 pg/mL (limit of quantification 50 pg/mL)	Analytical cycle (18 min) without pre-analysis

**Table 2:** Examples of biosensor technologies currently under development that satisfy the criteria for rapid testing (<10 min).

Biomarker	Technology	Transduction /Probe	LOD	Assay Time	Sample	Ref
NT-proBNP	Field effect transistor	Electrical /monoclonal Antibody	100 pg/mL	5 min	5 µm whole blood	[44]
	Field effect transistor	Electrical /Aptamer	0.83 pg/mL	5 min	4 µL plasma/serum	[45]
D-dimer	Graphene electrodes	Electrochemical (potentiometry) /Antibody	0.3 pg/mL	Dilution time + 15 s analysis time	Diluted serum	[46]
	Interdigitated electrodes on a compact disc	Electrical (capacitive) /Antibody	1 pg/mL	1 min after sample dilution	PBS	[47]

## Data Availability

Not applicable.

## References

[1] Abensur Vuillaume L., Leichle T., Le Borgne P., Grajoszex M., Goetz C., Voss P.L., Ougazzaden A., Salvestrini J.-P., d’Ortho M.-P. (2022). Relevant Biomarkers in Medical Practices: An Analysis of the Needs Addressed by an International Survey. J. Pers. Med..

[2] Thygesen K., Alpert J.S., White H.D., Jaffe A.S., Apple F.S., Galvani M., Joint ESC/ACCF/AHA/WHF Task Force for the Redefinition of Myocardial Infarction (2007). Universal definition of myocardial infarction. Eur. Heart J..

[3] (2017). Guidelines on Management of Acute Myocardial Infarction in Patients Presenting with ST-Segment Elevation. https://www.escardio.org/Guidelines/Clinical-Practice-Guidelines/Acute-Myocardial-Infarction-in-patients-presenting-with-ST-segment-elevation-Ma.

[4] Roffi M., Patrono C., Collet J.P., Mueller C., Valgimigli M., Andreotti F., Bax J.J., Borger M.A., Brotons C., Chew D.P. (2016). ESC Guidelines for the management of acute coronary syndromes in patients presenting without persistent ST-segment elevation: Task Force for the Management of Acute Coronary Syndromes in Patients Presenting without Persistent ST-Segment Elevation of the European Society of Cardiology (ESC). Eur. Heart J..

[5] White H.D. (2011). Pathobiology of troponin elevations: Do elevations occur with myocardialischemia as well as necrosis?. J. Am. Coll. Cardiol..

[6] Jaffe A.S., Babuin L., Apple F.S. (2006). Biomarkers in acute cardiac disease: The present andthe future. J. Am. Coll. Cardiol..

[7] Collet J.-P., Thiele H., Barbato E., Barthélémy O., Bauersachs J., Bhatt D.L., Dendale P., Dorobantu M., Edvardsen T., Folliguet T. (2020). Guidelines on Non-ST-Segment Elevation Acute Coronary Syndromes. ESC Guidelines. Eur. Heart J..

[8] Vaubourdolle M., Alvarez J.C., Barbé F., Beaudeux J.-L., Boissier E., Caillon H., Chatron P., Joly-Guillou M.-L., Mailloux A., Tournoys M.-H. (2018). Biologie d’urgence: Les recommandations 2018 de la SFBC. Ann. Biol. Clin..

[9] Brunel V., Larson T., Peschanski N., Cauliez B. (2012). Evaluation of haemolysis in emergency department samples requesting high sensitivity troponin T measurement. Ann. Clin. Biochem..

[10] Konstantinides S.V., Meyer G., Becattini C., Bueno H., Geersing G.J., Harjola V.P., Huisman M.V., Humbert M., Jennings C.S., Jiménez D. (2019). 2019 ESC Guidelines for the diagnosis and management of acute pulmonary embolism developed in collaboration with the European Respiratory Society (ERS): The Task Force for the diagnosis and management of acute pulmonary embolism of the European Society of Cardiology (ESC). Eur. Respir. J..

[11] Wells P., Anderson D.D., Rodger M., Forgie M., Kearon C., Dreyer J., Kovacs G., Mitchell M., Lewandowski B., Kovacs M.J. (2003). Evaluation of d-Dimer in the Diagnosis of Suspected Deep-Vein Thrombosis. New Engl. J. Med..

[12] Wells P.S., Anderson D.R., Rodger M., Stiell I., Dreyer J.F., Barnes D., Forgie M., Kovacs G., Ward J., Kovacs M.J. (2001). Excluding pulmonary embolism at the bedside without diagnosticimaging: Management of patients with suspected pulmonary embolism presenting to the emergency departmentby using a simple clinical model and D-dimer. Ann. Intern. Med..

[13] Freund Y., Chauvin A., Jimenez S., Philippon A.L., Curac S., Fémy F., Gorlicki J., Chouihed T., Goulet H., Montassier E. (2021). Effect of a Diagnostic Strategy Using an Elevated and Age-Adjusted D-Dimer Threshold on Thromboembolic Events in Emergency Department Patients with Suspected Pulmonary Embolism: A Randomized Clinical Trial. JAMA.

[14] Martinez-Rumayor A., Richards A.M., Burnett J.C., Januzzi J.L. (2008). Biologyof the natriuretic peptides. Am. J. Cardiol..

[15] Chen H.H., Burnett J.C. (1999). The natriuretic peptides in heart failure: Diagnostic and therapeutic potentials. Proc. Assoc. Am. Phys..

[16] Jourdain P., Lefèvre G., Oddoze C., Sapin V., Dievart F., Jondeau G., Meune C., Galinier M. (2009). NT-proBNP en pratique «De la biologie à la clinique». Ann. Cardiol. Angeiol..

[17] HAS (2010). Les Marqueurs Cardiaques Dans la Maladie Coronarienne et L’insuffisance Cardiaque en Médecine Ambulatoire. https://www.has-sante.fr/upload/docs/application/pdf/2010-09/rapport_marqueurs_cardiaques.pdf.

[18] Mant J., Doust J., Roalfe A., Barton P., Cowie M.R., Glasziou P., Mant D., McManus R.J., Holder R., Deeks J. (2009). Systematic review and individual patient data meta-analysis of diagnosis of heart failure, with modelling of implications of different diagnostic strategies in primary care. Health Technol. Assess..

[19] Dickstein K., Cohen-Solal A., Filippatos G., McMurray J.J., Ponikowski P., Poole-Wilson P.A., Strömberg A., van Veldhuisen D.J., Atar D., Hoes A.W. (2008). 2015 ESC guidelines for the diagnosis and treatment of acute and chronic heart failure 2008: The Task Force for the diagnosis and treatment of acute and chronic heart failure 2008 of the European Society of Cardiology. Developed in collaboration with the Heart Failure Association of the ESC (HFA) and endorsed by the European Society of Intensive Care Medicine (ESICM). Eur. J. Heart Fail..

[20] Wang C.S., FitzGerald J.M., Schulzer M., Mak E., Ayas N.T. (2005). Does this dyspneic patient in the emergency department have congestive heart failure?. JAMA.

[21] McDonagh T.A., Metra M., Adamo M., Gardner R.S., Baumbach A., Böhm M., Burri H., Butler J., Čelutkienė J., Chioncel O. (2021). 2021 ESC Guidelines for the diagnosis and treatment of acute and chronic heart failure: Developed by the Task Force for the diagnosis and treatment of acute and chronic heart failure of the European Society of Cardiology (ESC) With the special contribution of the Heart Failure Association (HFA) of the ESC. Eur. Heart J..

[22] Alawieh H., Chemaly T.E., Alam S., Khraiche M. (2019). Towards Point-of-Care Heart Failure Diagnostic Platforms: BNP and NT-proBNP Biosensors. Sensors.

[23] Ruppé E., Aubert C., Capeau J., Lefèvre G. (2005). Dosage du BNP et du NT-proBNP: Influence de l’étape pré-analytique. Immunoanal. Biol. Spec..

[24] Shimizu H., Aono K., Masuta K. (2001). Degradation of human brain natriu-retic peptide (BNP) by contact activation of blood coagulation system. Clin. Chim. Acta..

[25] Lewis R.A., Durrington C., Condliffe R., Kiely D.G. (2020). BNP/NT-proBNP in pulmonary arterial hypertension: Time for point-of-care testing?. Eur. Respir. Rev..

[26] Ray P., Birolleau S., Lefort Y., Becquemin M.H., Beigelman C., Isnard R., Teixeira A., Arthaud M., Riou B., Boddaert J. (2006). Acute respiratory failure in the elderly: Etiology, emergency diagnosis and prognosis. Crit Care..

[27] Jobs A., Simon R., de Waha S., Rogacev K., Katalinic A., Babaev V., Thiele H. (2018). Pneumonia and inflammation in acute decompensated heart failure: A registry-based analysis of 1939 patients. Eur. Heart J. Acute Cardiovasc. Care.

[28] Price S.P.E., Cullen L., Tavazzi G., Christ M., Cowie M.R., Cowie M.R., Maisel A.S., Masip J., Miro O., McMurray J.J. (2017). Expert consensus document: Echocardiography and lung ultrasonography for the assessment and management of acute heart failure. Nat. Rev. Cardiol..

[29] Wuerz R.C., Meador S.A. (1992). Effects of prehospital medications on mortality and length of stay in congestive heart failure. Ann. Emerg. Med..

[30] Claret P.G., Bobbia X., Roger C., Sebbane M., de La Coussaye J.E. (2015). Review of point-of-care testing and biomarkers of cardiovascular diseases in emergency and prehospital medicine. Acta Cardiol..

[31] Kobayashi M., Douair A., Duarte K., Jaeger D., Giacomin G., Bassand A., Jeangeorges V., Vuillaume L.A., Preud’homme G., Huttin O. (2020). Diagnostic performance of congestion score index evaluated from chest radiography for acute heart failure in the emergency department: A retrospective analysis from the PARADISE cohort. PLoS Med..

[32] Thévenot D.R., Toth K., Durst R.A., Wilson G.S. (2001). Electrochemical biosensors: Recommended definitions and classification. Biosens. Bioelectron..

[33] Ouyang M., Tu D., Tong L., Sarwar M., Bhimaraj A., Li C., Coté G.L., Carlo D.D. (2021). A review of biosensor technologies for blood biomarkers toward monitoring cardiovascular diseases at the point-of-care. Biosens. Bioelectron..

[34] Savonnet M., Rolland T., Cubizolles M., Roupioz Y., Buhot A. (2021). Recent advances in cardiac biomarkers detection: From commercial devices to emerging technologies. J. Pharm. Biomed. Anal..

[35] Nicu L., Leichle T. (2008). Biosensors and tools for surface functionalization from the macro- to the nanoscale: The way forward. J. Appl. Phys..

[36] Gervais L., de Rooij N., Delamarche E. (2011). Microfluidic Chips for Point-of-Care Immunodiagnostics. Adv. Mater..

[37] Land K.J., Boeras D.I., Chen X.S., Ramsay A.R., Peeling R.W. (2019). REASSURED diagnostics to inform disease control strategies, strengthen health systems and improve patient outcomes. Nat. Microbiol..

[38] Arlett J.L., Myers E.B., Roukes M.L. (2011). Comparative advantages of mechanical biosensors. Nat. Nanotechnol..

[39] Sarangadharan I., Regmi A., Chen Y.W., Hsu C.P., Chen C., Chang W.H., Lee Y.-G., Chyi J.-I., Shiesh S.-C., Lee G.-B. (2018). High sensitivity cardiac troponin I detection in physiological environment using AlGaN/GaN High Electron Mobility Transistor (HEMT). Biosens. Bioelectron..

[40] Liu J., Chen D., Wang P., Song G., Zhang X., Li Z., Wang Y., Wang J., Yang J. (2020). A microfabricated thickness shear mode electroacoustic resonator for the label-free detection of cardiac troponin in serum. Talanta.

[41] Kurita R., Yokota Y., Sato Y., Mizutani F., Niwa O. (2006). On-Chip Enzyme Immunoassay of a Cardiac Marker Using a Microfluidic Device Combined with a Portable Surface Plasmon Resonance System. Anal. Chem..

[42] Gachpazan M., Mohammadinejad A., Saeidinia A., Rahimi H.R., Ghayour-Mobarhan M., Vakilian F., Rezayi M. (2021). A review of biosensors for the detection of B-type natriuretic peptide as an important cardiovascular biomarker. Anal. Bioanal. Chem..

[43] Tasić N., Paixão T., Gonçalves L.M. (2020). Biosensing of D-dimer, making the transition from the central hospital laboratory to bedside determination. Talanta.

[44] Sarangadharan I., Huang S.-W., Kuo W.-C., Chen P.-H., Wang Y.-L. (2019). Rapid detection of NT-proBNP from whole blood using FET based biosensors for homecare. Sens. Actuators: B. Chem..

[45] Sinha A., Tai T.-Y., Li K.-H., Gopinathan P., Chung Y.-D., Sarangadharan I., Ma H.-P., Huang P.-C., Shiesh S.-C., Wang Y.-L. (2019). An integrated microfluidic system with field-effect-transistor sensor arrays for detecting multiple cardiovascular biomarkers from clinical samples. Biosens. Bioelectron..

[46] Nikoleli G.-P., Nikolelis D.P., Tzamtzis N., Psaroudakis N. (2014). A Selective Immunosensor for D-dimer Based on Antibody Immobilized on a Graphene Electrode with Incorporated Lipid Films. Electroanalysis.

[47] Li S., Jiang Y., Eda S., Wu J.J. (2019). Low-Cost and Desktop-Fabricated Biosensor for Rapid and Sensitive Detection of Circulating D-Dimer Biomarker. IEEE Sens. J..

[48] Istat Website. https://www.globalpointofcare.abbott/content/poc/en/pages/product-details/apoc/istat-ctnI-us.html.

[49] STAT Manual. https://www.lagaay.com/Catalogus/Product%20information/215720/Procedure%20Manual%20I-STAT.pdf.

[50] HAS. https://has-sante.fr/upload/docs/application/pdf/2010-11/fbuts_marcoeurs_necrose.pdf.

[51] Lim W.Y., Thevarajah T.M., Goh B.T., Khor S.M. (2019). Paper microfluidic device for early diagnosis and prognosis of acute myocardial infarction via quantitative multiplex cardiac biomarker detection. Biosens. Bioelectron..

[52] Bonnanni E., Dupont Y., Rerbal D. (2014). Biologie Délocalisée des Urgences. https://www.sfmu.org/upload/70_formation/02_eformation/02_congres/Urgences/urgences2014/donnees/pdf/086.pdf.

[53] Sarangadharan I., Wang S., Tai T., Pulikkathodi A.K., Hsu C., Chiang H.K., Liu L.Y.-M., Wang Y.-L. (2018). Risk stratification of heart failure from one drop of blood using hand-held biosensor for BNP detection. Biosens. Bioelectron..

[54] (2019). Arrêté du 4 Octobre 2019 Relatif à L’expérimentation d’un Parcours de Soins Intégrant la Biologie Délocalisée pour des Patients Chroniques sous AVK (Di@pason). https://www.legifrance.gouv.fr/jorf/id/JORFTEXT000039206940.

[55] Mani V., Durmus C., Khushaim W., Ferreira D.C., Timur S., Arduini F., Salama K.N. (2022). Multiplexed sensing techniques for cardiovascular disease biomarkers-A review. Biosens. Bioelectron..

